# Supplementary material for: Aurora Kinases Phosphorylate Lgl to Induce Mitotic Spindle Orientation in Drosophila Epithelia
Source: Curr Biol. 2015 Jan 5;25(1):61–8. doi: 10.1016/j.cub.2014.10.052 (PMC4291145; doi:10.1016/j.cub.2014.10.052)
Supplement: Document S1. Supplemental Experimental Procedures and Figures S1–S3 [file mmc1.pdf]

Current Biology, Volume *25*

Supplemental Information

Aurora Kinases Phosphorylate Lgl to Induce Mitotic Spindle Orientation in *Drosophila* Epithelia

Graham P. Bell, Georgina C. Fletcher, Ruth Brain, and Barry J. Thompson1

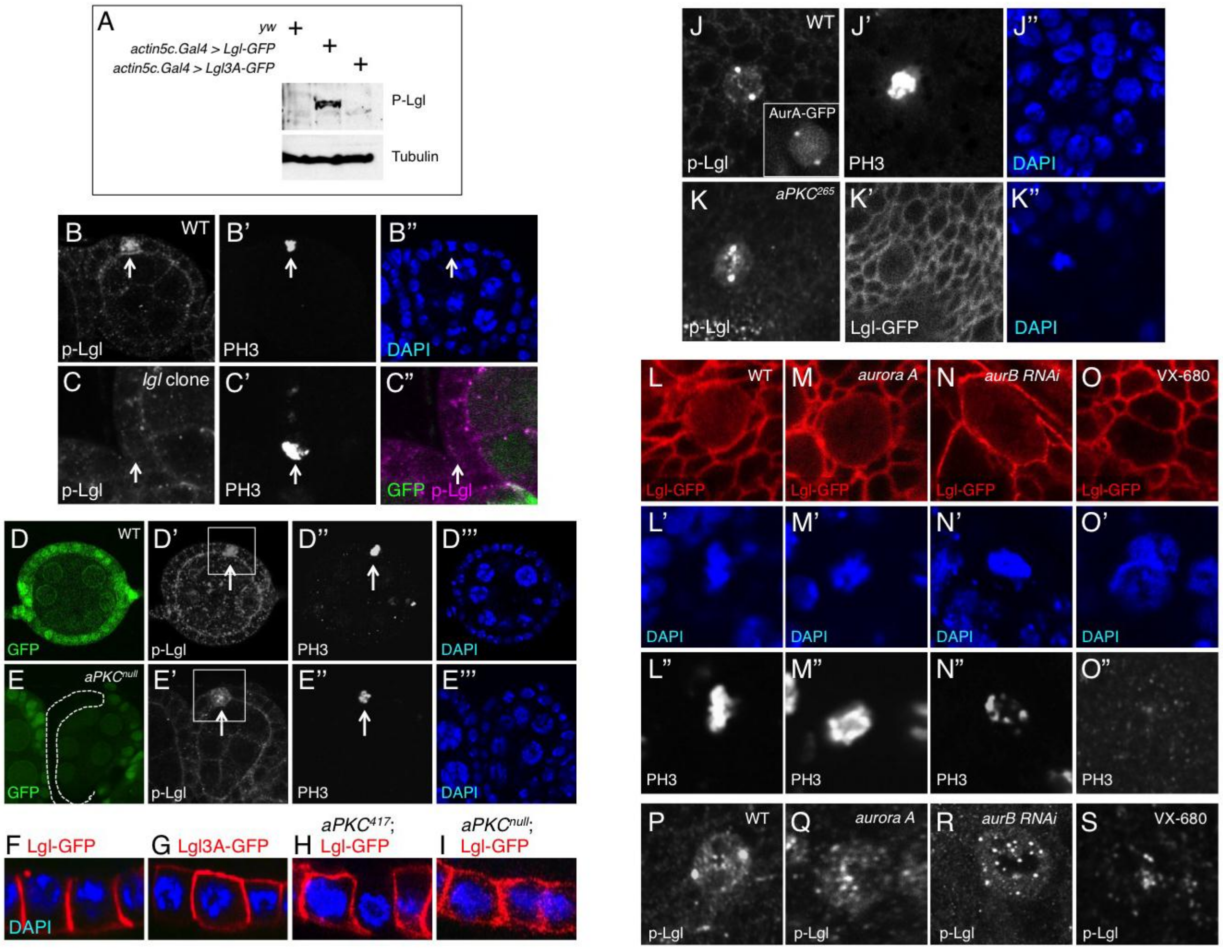

Figure S1. Mitotic phosphorylation of Lgl depends on Aurora A/B kinases, while aPKC kinase activity is only required to polarise Lgl during interphase.

(A) A phospho-Lgl antibody is detectable by Western blotting upon expression of Lgl-GFP, but not Lgl3A-GFP or in *yw* specimens.
(B-C) Phospho-Lgl strongly accumulates in the cytoplasm of mitotic cells in the follicle cell epithelium of control (B) but not *lgl*^{4} mutant clones (C).
(D-E) Phospho-Lgl strongly accumulates in the cytoplasm of mitotic cells in both control (D) and *aPKC*^{K06403} null mutant (E) follicle cells.
(F) Lgl-GFP control showing normal polarisation in the follicle cell epithelium.
(G) Lgl3A-GFP is not polarised and localises around the entire plasma membrnane.
(H) Lgl-GFP is no longer polarised in *aPKC*^{417} kinase-dead mutant MARCM clones.
(I) Lgl-GFP is no longer polarised in *aPKC*^{K06403} null mutant MARCM clones.
(J-K) Phospho-Lgl accumulates in the cytoplasm of mitotic cells in the wing imaginal discs of both control (J) and *aPKC*^{265} kinase-dead mutant MARCM clones (K). Note that AuroraA-GFP localisation is almost identical to p-Lgl antibody staining (inset).
(L-O) Lgl can still relocalise to the cytoplasm in control (L), *aurA*$^{87Ac\text{-}3}$*/Df* mutants (M) or *en.Gal4 UAS-aurB-RNAi* (N), but not upon treatment with the dual Aurora kinase inhibitor VX-680 (O). PH3 staining was not affected by mutation of *aurA* (M), but was strongly reduced in both *aurB* RNAi (N) and VX-680 treated discs (O).
(P-S) Phospho-Lgl accumulation in mitosis (P) can still be detected in either *aurA*$^{87Ac\text{-}3}$*/Df* mutants (Q) or *en.Gal4 UAS-aurB-RNAi* (N), but not upon treatment with the dual Aurora kinase inhibitor VX-680 (S). P, Q and S are the same cell as in L, M and O.

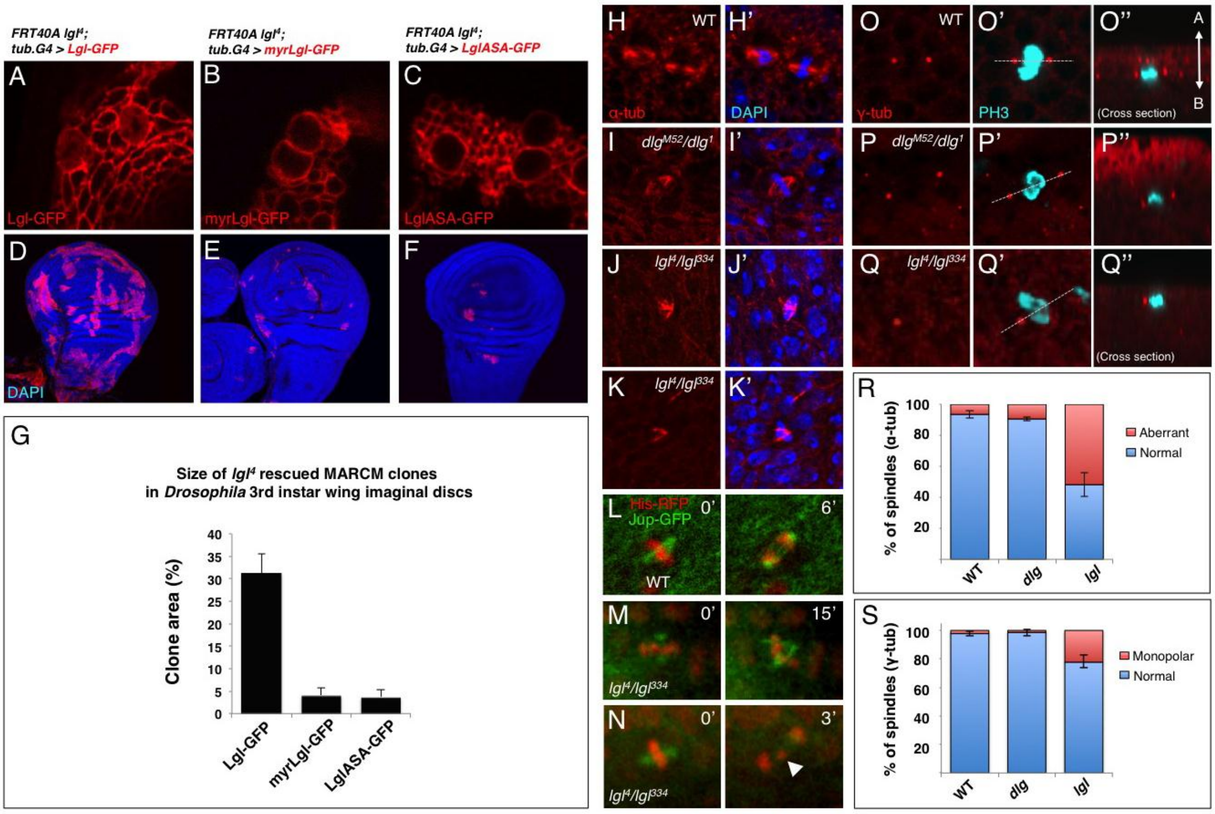

Figure S2. A mitotic role for Lgl in clonal growth and spindle formation in the wing imaginal disc epithelium.

(A-G) MARCM clones of *lgl*^{4} mutant cells expressing Lgl-GFP (A), myrLgl-GFP (B) or LglASA-GFP (C). Note that myr-Lgl-GFP and LglASA-GFP do not relocalise efficiently to the cytoplasm at mitosis. Whilst clones of *lgl*^{4} mutant cells expressing Lgl-GFP grow normally (D), clones of *lgl*^{4} mutant cells expressing myrLgl-GFP (E) or LglASA-GFP (F) grow to a small size, suggesting a mitotic role for Lgl that is required to prevent apoptosis or promote cell proliferation. (G) Quantification of clone sizes in D-F.

(H-K) Mitotic spindles form normally in wild type (H) and *dlg*^{M52}/*dlg*^{1} (I) wing imaginal discs. Mitotic spindles form abnormally in *lgl*^{4}/*lgl*^{334} mutant wing imaginal discs (J,K). α-tubulin (red) and DAPI (blue) are stained.

(L-N) Live imaging of wild type (L) or *lgl*^{4}/*lgl*^{334} (M, N) mutant discs reveals similar defects in spindle organisation to fixed samples. Note the similarity in spindle phenotype between (K) and (M). Note the unsuccessful chromosome segregation in (N).

(O-Q) Mitotic spindles in wild type tissues are always bipolar, and are oriented parallel to the plane of the tissue (O). *dlg*^{M52}/*dlg*^{1} mutant discs show spindles misaligned with the plane of the tissue, but no defects in centrosome number (P). *lgl*^{4}/*lgl*^{334} mutant discs show some monopolar spindles, in addition to defects in spindle orientation (Q).

(R-S) Quantification of mitotic spindle defects in wild type, *dlg* mutant and *lgl* mutant discs by α-tubulin (R) or γ□tubulin (S) staining.

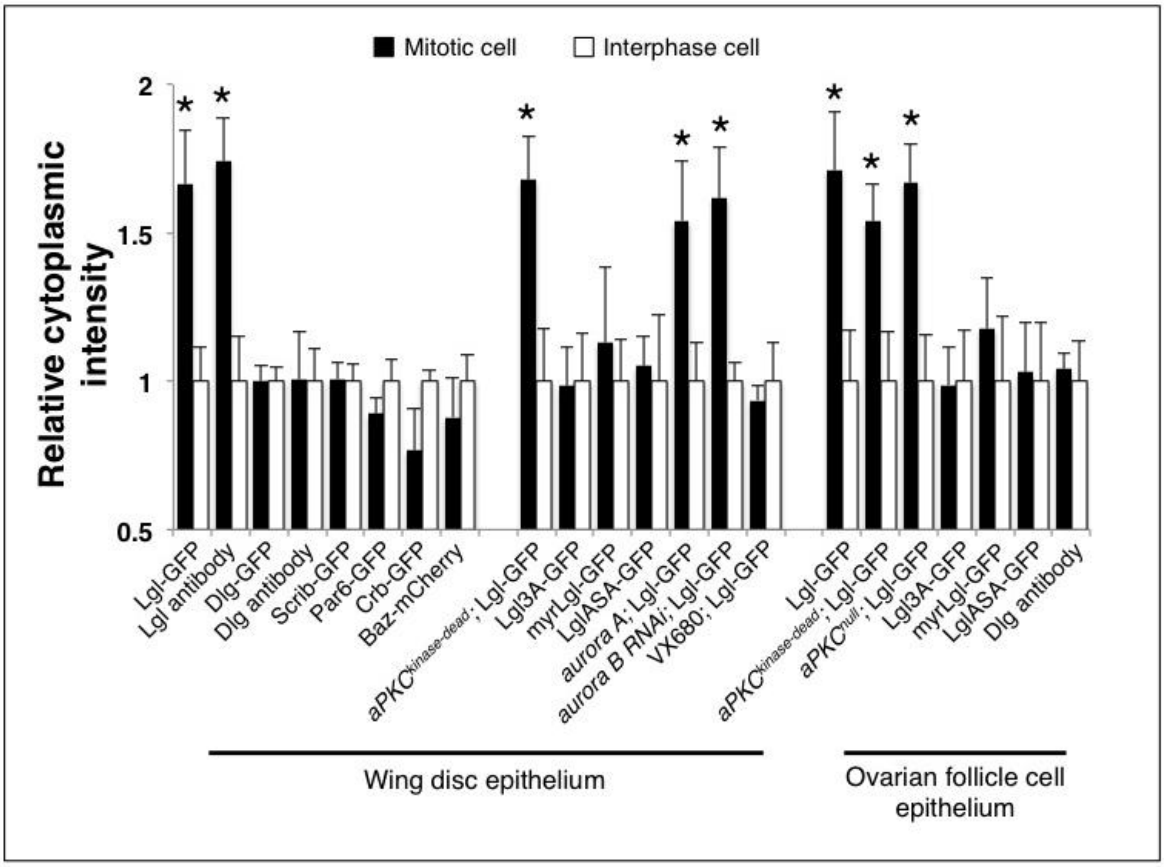

Figure S3. Quantification of intensity measurements.

Supplemental Experimental Procedures

Drosophila genetics

Transgenes driven by a UAS-promoter were induced by crossing lines to the *MS1096.Gal4, en.Gal4,* or *hh.Gal4* drivers for wing disc expression; either *tub.Gal4* or *tj.Gal4* for follicle cell expression; *wor.Gal4* for neuroblasts; and *actin5c.Gal4* for whole animal expression. Mitotic clones were generated using the FLP/FRT system by heat-shocking flies at 37 degrees for 1hr during early larval development, and marked negatively (absence of GFP) or positively (presence of GFP; MARCM). Expression of *UAS-Lgl-GFP, UAS-myrLgl-GFP,* or *UAS-LglASA-GFP* in *FRT* wild-type or *FRT* mutant backgrounds was achieved using the MARCM system. Clones were induced at the same time in cultures of controlled density in each experiment. For wing disc or brain clonal analysis, third instar larvae were dissected; for follicle cell analysis, heat-shocked larvae were grown to adult, and females dissected approximately 4 days after eclosion.

Antibody staining

Wing imaginal discs or brains dissected from third instar larvae, or ovaries dissected from adult females, were fixed for 30 mins in cold PBS containing 4% paraformaldehyde. Samples were washed in PBS supplemented with 0.1% Triton-X100 (PBT), blocked with 0.1% Bovine Serum Albumin (PBT + BSA) (and 5% Normal Goat Serum for ovaries), and stained with primary and fluorescently conjugated secondary antibodies.

Primary antibodies used were:

Rabbit anti-phospho LLGL1/2 (p-Lgl) (1:250, Abgent), mouse anti-phosphohistone H3 (PH3) (1:1000, Abcam), rabbit anti-phosphohistone H3 (PH3) (1:1000, Millipore), mouse anti-α tubulin (1:500, Sigma), mouse anti-Dlg (1:250 DSHB), rabbit anti-aPKC (1:250, Santa Cruz), rabbit anti-γ tubulin (1:500, Sigma), mouse anti-Miranda (1:50, gift from A. Gould), mouse anti-GFP (1:250, Roche), rabbit anti-

centrosomin (CNN) (1:1000, gift from J. Raff).

Secondary antibodies (all from Molecular Probes, Invitrogen), were used at 1:500 for 2 hours prior to multiple washes in PBT and staining with DAPI at 1µg/ml for 10-minutes, before mounting on slides in Vectashield (Vector labs). Images were taken on a Leica SP5 confocal and processed with Adobe Photoshop and ImageJ.

Live imaging
Live imaging of *ex vivo* cultured wing discs was performed with a Zeiss 780 confocal microscope. Briefly, discs were cultured in Shields and Sang M3 media (Sigma) containing 2% fetal bovine serum, 10 µg/mL streptomycin/penicillin (Invitrogen), 10 mU/L insulin, 0.1 µg/mL ecdysone (Sigma) and 2.5% methyl cellulose (Sigma), and imaged in a 35mm-Fluorodish. *Z*-stacks were taken at 1-µm intervals, with total thickness of 10–30 µm. *Z*-stacks were typically scanned at 2-min intervals for up to 3 h. Images were projected and time points were collated using Zen software.

For drug treatment, inverted larvae were incubated in culture media as described above (without methyl cellulose) with 2µM VX-680/Tozasertib (Selleck BioChem) for 30-60 minutes, then transferred to media as described above for imaging.

In vitro kinase assay
For *in vitro* kinase assays, HPLC purified peptide substrates were incubated with either 200ng recombinant human Aurora A (Promega) or 80ng recombinant human Aurora B (Promega) for 30 min at 30°C in Reaction Buffer A (Promega) (200mM Tris-HCL (pH 7.5), 100mM $MgCl_2$, 0.5 µg/µl BSA, 50µM DTT) containing 10µM cold adenosine triphosphate (ATP) and 3µCi of ϒ-P32 ATP (Perkin Elmer). Samples were blotted on P81 phosphocellulose paper (Millipore) and washed 3 x 10 min in 1% phosphoric acid, then once in acetone. Incorporation of γ-P^{32} was quantified in counts per minute (cpm) by liquid scintillation (Beckman LS 6500).

Peptides used in this study were:

Lgl	LSRRKSFKKSLRESFRKLR (2422.91 Da)
Lgl 3A (S656A, S660A, S664A)	LSRRKAFKKALREAFRKLR (2374.91 Da)
Lgl ASA (S656A, S664A)	LSRRKAFKKSLREAFRKLR (2390.91 Da)
Lgl SAS (S660A)	LSRRKSFKKALRESFRKLR (2406.91 Da)
Lgl AAS (S656A, S660A)	LSRRKAFKKALRESFRKLR (2390.91 Da)
Lgl ASS (S656A	LSRRKAFKKSLRESFRKLR (2406.91 Da)
CENP-A	MGPRRRSRKPEAPRRRSPSP (2374.77 Da)
CENP-A S7A	MGPRRRARKPEAPRRRSPSP (2358.77 Da)

Peptides were diluted with deionised water to working dilutions (1mg/ml) and stored at -20°C.

Western Blotting
Actin5c.Gal4/UAS-Lgl-GFP or *Actin5c.Gal4/UAS-Lgl3A-GFP* third instar larvae were dissected in cold PBS, and homogenised in sample buffer (50mM Tris pH7.5, 150mM NaCL, 1% Triton X-100, 1µM EGTA) supplemented with protease inhibitor cocktail (Roche), and phosphatase inhibitor cocktail (Roche) where appropriate. Samples were incubated for 30 minutes: with 2000 units/ml λ protein phosphatase (New England Biolabs) at 30°C, with 2µM VX-680, or on ice. SDS sample buffer and reducing agent (NuPage, Invitrogen) were added, and samples heated at 70°C for 10 minutes before SDS Page and Western Blotting (NuPage, Invitrogen). Nitrocellulose membranes were probed with rabbit anti-phospho-LLGL1/2 (1:100, Abgent) and mouse anti-Tubulin (1:1000, DSHB), and after several washes in TBST, probed with HRP-conjugated secondary antibodies (1:10,000, Thermo Scientific), with proteins detected by chemiluminescence.
