# Supplementary figures and images for: Aurora Kinases Phosphorylate Lgl to Induce Mitotic Spindle Orientation in Drosophila Epithelia
Source: Curr Biol. 2015 Jan 5;25(1):61–8. doi: 10.1016/j.cub.2014.10.052 (PMC4291145; doi:10.1016/j.cub.2014.10.052)

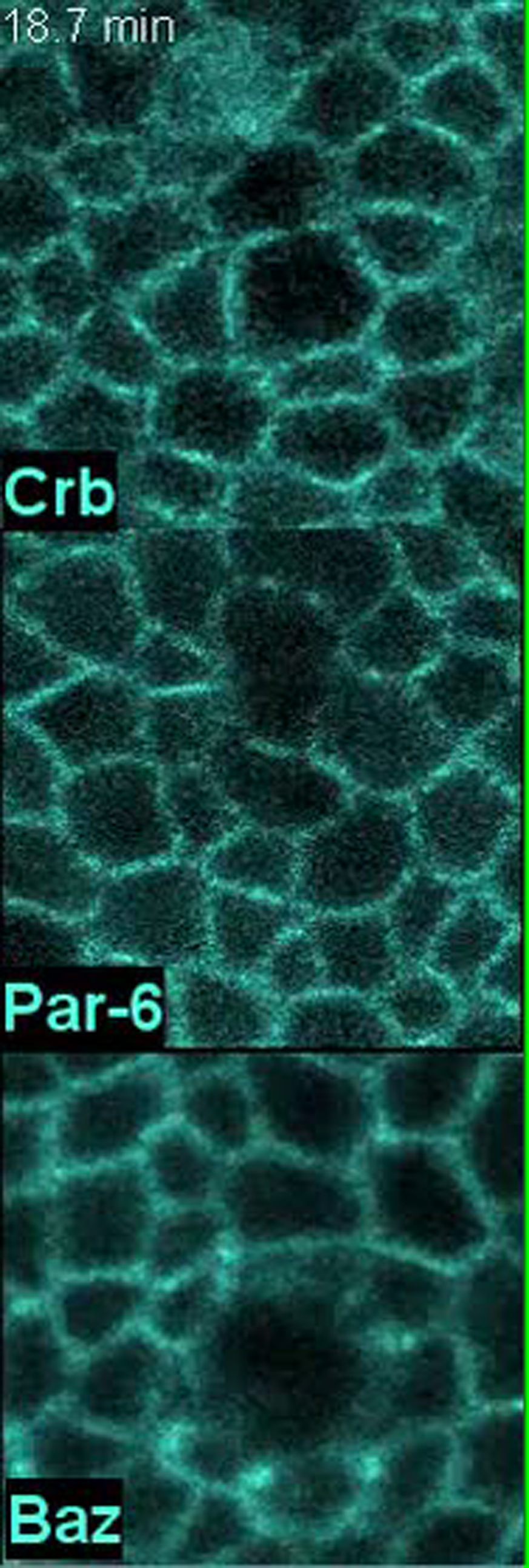

Supplement: Movie S1. Live Imaging of Apical Polarity Determinants during Mitosis in the Wing Disc, Related to Figure 1 [file mmc2.jpg]

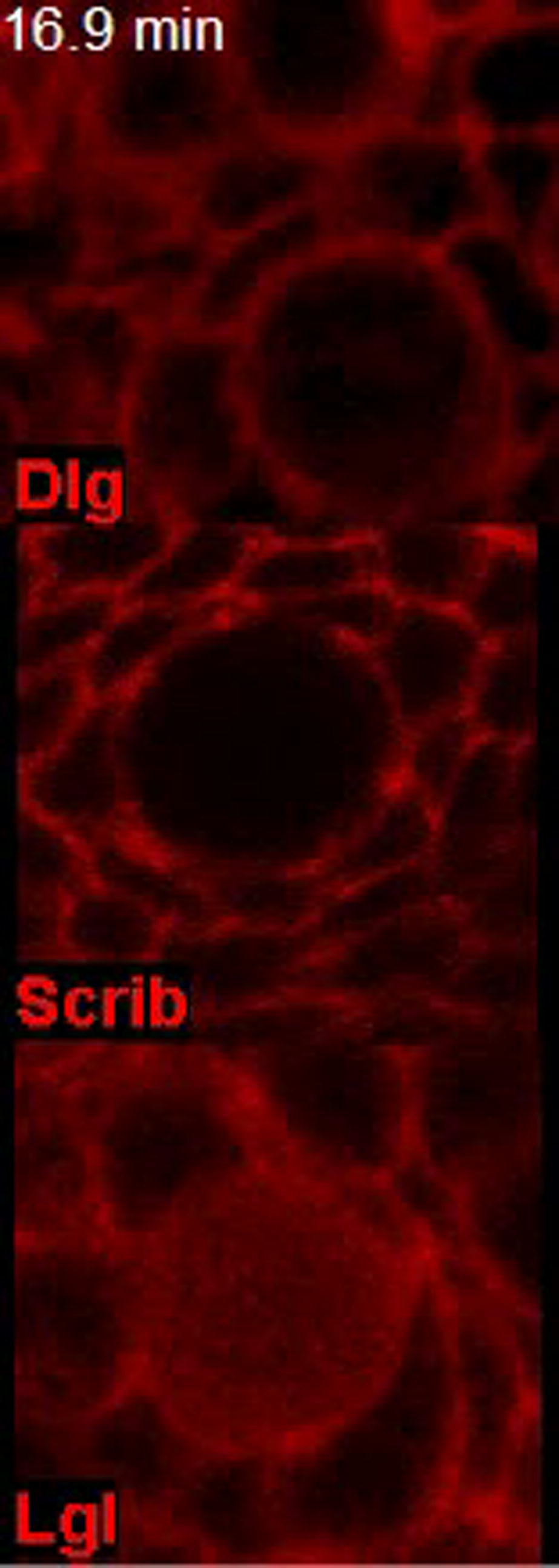

Supplement: Movie S2. Live Imaging of Basolateral Polarity Determinants during Mitosis in the Wing Disc, Related to Figure 1 [file mmc3.jpg]

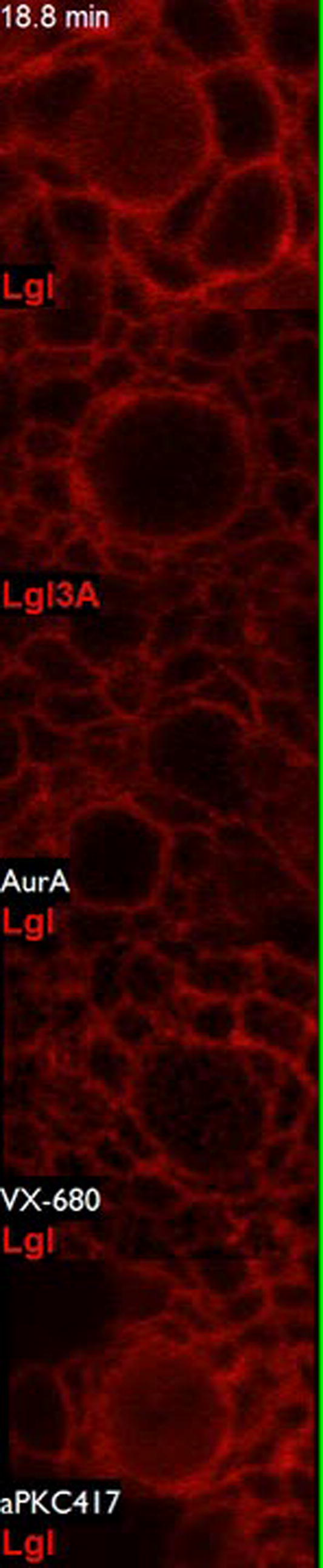

Supplement: Movie S3. Aurora Kinases Phosphorylate Lgl to Promote Its Relocalization to the Cytoplasm in Mitosis, Related to Figure 1 [file mmc4.jpg]
