# Supplementary material for: Aurora Kinases Phosphorylate Lgl to Induce Mitotic Spindle Orientation in Drosophila Epithelia
Source: Curr Biol. 2015 Jan 5;25(1):61–8. doi: 10.1016/j.cub.2014.10.052 (PMC4291145; doi:10.1016/j.cub.2014.10.052)
Supplement: Document S2. Article plus Supplemental Information [file mmc5.pdf]

Current Biology 25, 61–68, January 5, 2015 ©2015 The Authors http://dx.doi.org/10.1016/j.cub.2014.10.052

Report

Aurora Kinases Phosphorylate Lgl to Induce Mitotic Spindle Orientation in *Drosophila* Epithelia

Graham P. Bell,[1] Georgina C. Fletcher,[1] Ruth Brain,[1] and Barry J. Thompson[1],*
[1]Epithelial Biology Laboratory, Cancer Research UK, London Research Institute, Lincoln's Inn Fields, London WC2A 3LY, UK

Summary

The Lethal giant larvae (Lgl) protein was discovered in *Drosophila* as a tumor suppressor in both neural stem cells (neuroblasts) and epithelia. In neuroblasts, Lgl relocalizes to the cytoplasm at mitosis, an event attributed to phosphorylation by mitotically activated aPKC kinase and thought to promote asymmetric cell division. Here we show that Lgl also relocalizes to the cytoplasm at mitosis in epithelial cells, which divide symmetrically. The Aurora A and B kinases directly phosphorylate Lgl to promote its mitotic relocalization, whereas aPKC kinase activity is required only for polarization of Lgl. A form of Lgl that is a substrate for aPKC, but not Aurora kinases, can restore cell polarity in *lgl* mutants but reveals defects in mitotic spindle orientation in epithelia. We propose that removal of Lgl from the plasma membrane at mitosis allows Pins/LGN to bind Dlg and thus orient the spindle in the plane of the epithelium. Our findings suggest a revised model for Lgl regulation and function in both symmetric and asymmetric cell divisions.

Introduction

The Lethal giant larvae (Lgl) protein was first discovered as a tumor suppressor in the fruit fly *Drosophila* [1–3]. Mutants in *lgl* produce tumors in *Drosophila* epithelial tissues and in the brain [1]. The Lgl protein was later shown to function in cell polarity, which is disrupted in *lgl* mutant tumors [4–6]. In both epithelial cells and neuroblasts (neural stem cells), Lgl acts to restrict the localization of apical polarity determinants to the apical membrane domain [4–7]. These apical determinants include the atypical protein kinase C (aPKC), Par6, Cdc42, and Bazooka/Par3 [8–10]. Thus, Lgl is thought to have a common function in epithelia and neuroblasts.

Despite these commonalities between epithelial polarity and neuroblast polarity, recent results suggested that Lgl may be regulated differently in the two cell types. In epithelial cells, which are constitutively polarized and divide symmetrically, Lgl localizes to the basolateral membrane because it is excluded from the apical membrane upon phosphorylation by aPKC [11–13]. In neuroblasts, which only polarize at mitosis and divide asymmetrically, Lgl is initially removed apically but is then removed from the entire plasma membrane so that it relocalizes to the cytoplasm during mitosis [11, 14]. This relocalization of Lgl to the cytoplasm was proposed to be dependent on its phosphorylation by aPKC and to be important for asymmetric cell division because it would allow aPKC to act upon cell-fate determinants such as Miranda and Numb [11, 14]. Such a model is plausible in neuroblasts because removal of Lgl from the membrane requires three putative aPKC phosphorylation sites and because aPKC becomes activated and polarized specifically in mitosis, after activation of the mitotic Aurora A kinase, just before Lgl is removed from the plasma membrane [11, 14].

Here, we show that Lgl also relocalizes to the cytoplasm during mitosis in both the *Drosophila* wing disc and follicle cell epithelia. Since aPKC is thought to be constitutively active at the apical domain of epithelial cells, it is not clear how aPKC could account for the sudden mitotic relocalization of Lgl. Instead, we show that mitotic kinases Aurora A and B directly phosphorylate Lgl to mediate its relocalization during mitosis. We then construct a mutant form of Lgl that can be phosphorylated by aPKC, but not by Aurora kinases, and investigate its localization and function in vivo. Our findings suggest a revised model of Lgl regulation and function in both epithelia and neuroblasts. Note that similar findings were recently reported by Carvalho et al. [15].

Results and Discussion

Lgl Becomes Cytoplasmic during Mitosis in the *Drosophila* Wing Epithelium

The localization of polarity determinants during mitosis has been well characterized in asymmetrically dividing neuroblasts of *Drosophila*. In contrast, how polarity determinants are localized in epithelial cells during mitosis remains poorly understood. We therefore live imaged several fluorescently tagged apical and basolateral polarity determinants during epithelial mitosis in the *Drosophila* larval wing imaginal disc. We find that most GFP-tagged polarity determinants—including Crb, Par6, Baz, Dlg, and Scrib—retain their polarized localization through mitosis, whereas Lgl-GFP relocalizes to the cytoplasm (Figures 1A–1F). The relocalization of Lgl to the cytoplasm is complete because no Lgl-GFP can be detected on the plasma membrane in mitotic cells at the edge of a clone expressing Lgl-GFP (Figures 1G and 1H; Movies S1 and S2 available online). Similar observations of Lgl-GFP were previously noticed in the *Drosophila* embryonic epithelium [16]. Quantification is provided in Figure S3.

Mitotic Relocalization of Lgl Does Not Depend on aPKC Kinase Activity

Since the aPKC kinase was proposed to be responsible for mitotic relocalization of Lgl in neuroblasts, we tested whether aPKC kinase activity was required for this event in epithelial cells. We find that clones of *aPKC*^{417} kinase-dead mutant cells can still relocalize Lgl-GFP to the cytoplasm at mitosis in the wing imaginal disc, and similar results were obtained with other kinase-dead alleles (Figure 1I; Movie S3). We further find that an anti-phospho-Lgl antibody strongly stains both wild-type and *aPKC*-null mutant mitotic cells (Figure S1). In contrast, mutation of the three potential aPKC phosphorylation sites in Lgl (Lgl3A-GFP) completely prevents relocalization to the cytoplasm (Figure 1J) [11, 12, 14]. These results show that mitotic relocalization of Lgl does not depend on phosphorylation of

*Correspondence: barry.thompson@cancer.org.uk
This is an open access article under the CC BY license (http://creativecommons.org/licenses/by/3.0/).
CrossMark

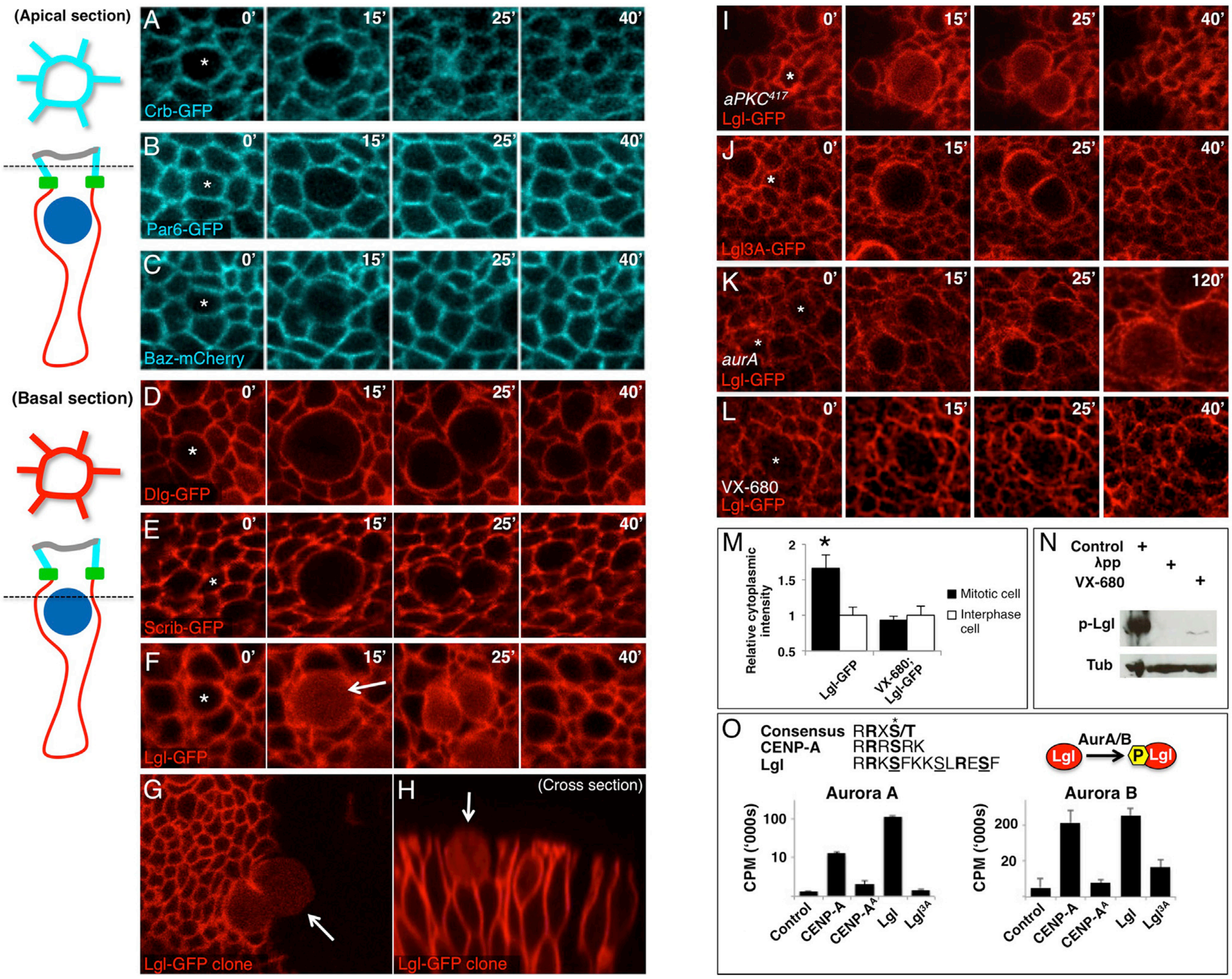

Figure 1. Aurora Kinases Phosphorylate Lgl to Relocalize It to the Cytoplasm during Mitosis in the Wing Epithelium

(A–C) Live imaging of third instar wing imaginal discs. Fluorescently tagged Crumbs (Crb-GFP; A) and Par-6 (Par-6-GFP; B) remain apically localized during mitosis (apical section; diagrammed on the left). Baz-mCherry is partially downregulated but remains apical (C).

(D and E) Fluorescently tagged Discs-large (Dlg-GFP; D) and Scribble (Scrib-GFP; E) remain basolaterally localized during mitosis (basal section; diagrammed on the left).

(F–H) Fluorescently tagged Lethal giant larvae (Lgl-GFP) becomes cytoplasmic at mitosis (basal section; diagrammed on the left): note the complete relocalization at mitosis with no membrane staining (G).

(I) Lgl-GFP relocalizes to the cytoplasm at mitosis normally in an *aPKC*^{417} kinase-dead MARCM clone in wing disc epithelia, indicating that aPKC phosphorylation is not responsible for regulating Lgl at mitosis. Other kinase-dead alleles of aPKC gave similar results (not shown).

(J) Phosphomutant Lgl3A-GFP fails to relocalize to the cytoplasm during mitosis in wing disc epithelia.

(K) Relocalization of Lgl-GFP to the cytoplasm in mitosis is strongly delayed in *aurA*$^{87Ac-3}$*/Df* mutants. Similar results were obtained with other *aurora A* mutant alleles or with AurA RNAi (not shown).

(L) Relocalization of Lgl-GFP to the cytoplasm in mitosis is blocked upon treatment of the epithelium with the Aurora A/B inhibitor VX-680.

(M) Quantification of control and VX-680 treated wing discs expressing Lgl-GFP.

(N) Western blotting analysis of *actin5c.Gal4 UAS-Lgl-GFP* wing discs showing absence of a strong phosphorylated Lgl band upon treatment of samples with λ-phosphatase or treatment of the epithelium with VX-680 prior to sample preparation.

(O) Aurora A and B directly phosphorylate the Lgl phosphosite motif in an in vitro kinase assay. CENP-A is a positive control substrate that is known to be phosphorylated by both Aurora A and B.

Error bars indicate 1 SD from the mean. See also Figures S1 and S3 and Movies S1, S2, and S3.

Lgl by aPKC and suggest that a different kinase must phosphorylate Lgl on at least one of the three key serine residues that control its association to the plasma membrane.

Aurora Kinases Directly Control Relocalization of Lgl at Mitosis in the Wing Epithelium

The mitotic Aurora A and B kinases are strong candidates to phosphorylate Lgl in mitosis, since they are well known to be activated specifically in mitosis and to have a consensus motif (RX[S/T]) that is found within the Lgl tripartite phosphorylation sequence [17]. Aurora A and B are thought to have some distinct targets because the *aurA* gene is required for timely entry into mitosis whereas the *aurB* gene is required for cytokinesis [17]. However, these kinases are also highly similar to one another and can have common targets, such as the centromere protein CENP-A, suggesting that they may have

some redundant functions [18, 19]. Compared to wild-type wing epithelia expressing Lgl-GFP, the mitotic relocalization of Lgl-GFP is dramatically delayed in *aurA* mutant clones (Figure 1K; Movie S3). We used the VX-680 compound, which can inhibit the kinase activity of both Auroras, to test redundancy of the Auroras. Acute treatment of wing epithelia in culture with VX-680 leads to a complete blockage of Lgl-GFP relocalization, even in cells that have already rounded up in preparation for mitosis (Figures 1L and 1M; Movie S3). Phosphorylation of Lgl as determined by western blotting with the p-Lgl antibody also reveals that inhibition of Auroras with VX-680 strongly reduces Lgl phosphorylation (Figure 1N). To test whether Aurora A and B can directly phosphorylate Lgl, rather than acting through another kinase, we performed in vitro kinase assays with purified Aurora A and B kinases. We find that both Aurora kinases can directly phosphorylate the key Lgl tripartite phosphorylation motif, but not when the three serines are mutated to alanine (Lgl3A; Figure 1O). These results show that Aurora A and B kinases can directly phosphorylate Lgl and suggest that this phosphorylation event is required for relocalization of Lgl to the cytoplasm during mitosis.

Generation of an Aurora-Insensitive Form of Lgl

Our findings raise the question of which of the three serines is in fact phosphorylated by Aurora kinases. Only the first and third serines match the RX[S/T] consensus motif for Auroras, suggesting that the middle site may be an exclusive site for aPKC (Figure 2A). Consistently, mutation of only the first and third serines (LglASA) abolishes Aurora A phosphorylation, whereas mutation of the middle serine (LglSAS) does not affect Aurora A phosphorylation in vitro (Figure 2B). When expressed in wing or follicle cell epithelia, the Aurora-insensitive LglASA-GFP completely fails to relocalize to the cytoplasm during mitosis (Figures 2C and 2D). However, LglASA-GFP is still polarized normally during interphase, unlike Lgl3A-GFP (Figures 2E–2G). Together, the above results indicate that regulation of Lgl during cell polarization and mitosis is mediated by distinct kinases: aPKC and Aurora A/B, respectively (Figure 2H).

Aurora Kinases Phosphorylate Lgl to Trigger Mitotic Spindle Orientation in Wing Disc Epithelia

We next sought to establish the relative functional importance of Aurora kinase phosphorylation of Lgl in vivo. To do so, we performed rescue experiments in *lgl* mutant clones with wild-type Lgl-GFP, with Aurora-insensitive LglASA-GFP, or with a membrane-tethered myristylated Lgl-GFP (myrLgl-GFP) that cannot be removed from the plasma membrane by either aPKC or Auroras. We find that Lgl-GFP rescues cell polarity in *lgl* mutant wing discs, as expected (Figure 2I). In contrast, both LglASA-GFP and myrLgl-GFP rescue cell polarity (Figures 2J–2M) but reveal mitotic spindle orientation defects (Figures 2N and 2O). The degree of spindle orientation failure is comparable to that of *pins* or *mud* mutants, being slightly stronger than the *pins* phenotype but slightly weaker than the *mud* phenotype (Figure 2O). These results indicate that phosphorylation of Lgl by Aurora kinases is required for normal mitotic spindle orientation in the wing epithelium. We also observe an additional role for Lgl in promoting spindle formation and clonal growth in this tissue (Figure S2).

Aurora Kinases Phosphorylate Lgl to Trigger Mitotic Spindle Orientation in the Follicle Cell Epithelium

To confirm our findings in another epithelial tissue, we examined the follicle cell epithelium that surrounds the developing egg chamber. We find that Lgl-GFP relocalizes to the cytoplasm during mitosis in follicle cells (Figures 3A and 3G). Relocalization is not affected in aPKC kinase-dead or null mutant clones (Figures 3B and 3C, G). However, Lgl3A-GFP, myrLgl-GFP, and LglASA-GFP all fail to relocalize to the cytoplasm during mitosis, indicating that phosphorylation by Aurora A/B kinases is required to relocalize Lgl to the cytoplasm (Figures 3D–3G and S3). Like wild-type Lgl-GFP, both the myrLgl-GFP and LglASA-GFP constructs rescue cell polarity in *lgl* mutant follicle cell clones (Figures 3H–3K). However, unlike wild-type Lgl-GFP, neither the myrLgl-GFP nor the LglASA-GFP construct was able to restore normal mitotic spindle orientation in *lgl* mutant clones in the follicle cell epithelium (Figures 3L–3T). Together, these results indicate that relocalization of Lgl to the cytoplasm upon phosphorylation by Aurora kinases is necessary for mitotic spindle orientation in the follicle cell epithelium.

Aurora-Mediated Phosphorylation of Lgl Is Dispensable in Neuroblasts

Since the original proposal that aPKC phosphorylates Lgl to relocalize it to the cytoplasm was based on results in neuroblasts [11, 14], we decided to re-examine regulation of Lgl in this system (Figure 4A). In larval brain neuroblasts, we find that Lgl-GFP behaves as previously described [11, 14], being removed from the entire plasma membrane during mitosis (Figure 4B). Aurora-insensitive LglASA-GFP behaves differently, being removed apically but remaining localized at the basal plasma membrane during mitosis (Figure 4C). Finally, myrLgl-GFP remains localized to the entire plasma membrane during mitosis and recruits aPKC along with it (Figure 4D). These results indicate that aPKC kinase activity is normally responsible for removing Lgl from the apical domain, whereas activation of Aurora A/B kinases removes Lgl from the entire plasma membrane during mitosis—as in epithelial cells.

We next examined the ability of our different Lgl constructs to rescue *lgl* mutant clones in neuroblasts. Compared to wild-type neuroblasts, loss of *lgl* in clones leads to ectopic activation of aPKC, which then causes relocalization of Miranda to the cytoplasm and symmetric cell division (Figures 4E and 4F). Expression of Lgl-GFP or LglASA-GFP in *lgl* mutant clones was able to rescue polarization of Miranda and asymmetric cell division, as well as spindle orientation (Figures 4G and 4H). Thus, Aurora-mediated relocalization of Lgl to the cytoplasm at mitosis is not essential for asymmetric cell division because Lgl remains polarized at the plasma membrane due to the action of aPKC, which is then sufficient to allow polarization of other substrates such as Miranda. When polarization of Lgl was completely prevented by expression of myrLgl-GFP in *lgl* mutant clones, it resulted in an inhibition of aPKC activity, as revealed by spreading of Miranda around the entire plasma membrane (Figure 4I). Finally, we find that mitotic spindle orientation is normal in *lgl* mutant neuroblasts rescued by expression of Lgl-GFP, LglASA-GFP, or myrLgl-GFP (Figure 4J). These results indicate that Aurora-mediated relocalization of Lgl to the cytoplasm at mitosis is dispensable for asymmetric cell division and spindle orientation in neuroblasts.

Our results suggest a revised model for Lgl regulation and function in both symmetric and asymmetric cell divisions. In the case of asymmetric cell divisions, our findings revise the prevailing notion that mitotic activation of aPKC is solely responsible for displacing Lgl into the cytoplasm [11, 14]. Instead, aPKC phosphorylation of Lgl can only remove Lgl from the apical domain of these cells. Aurora A/B kinases are

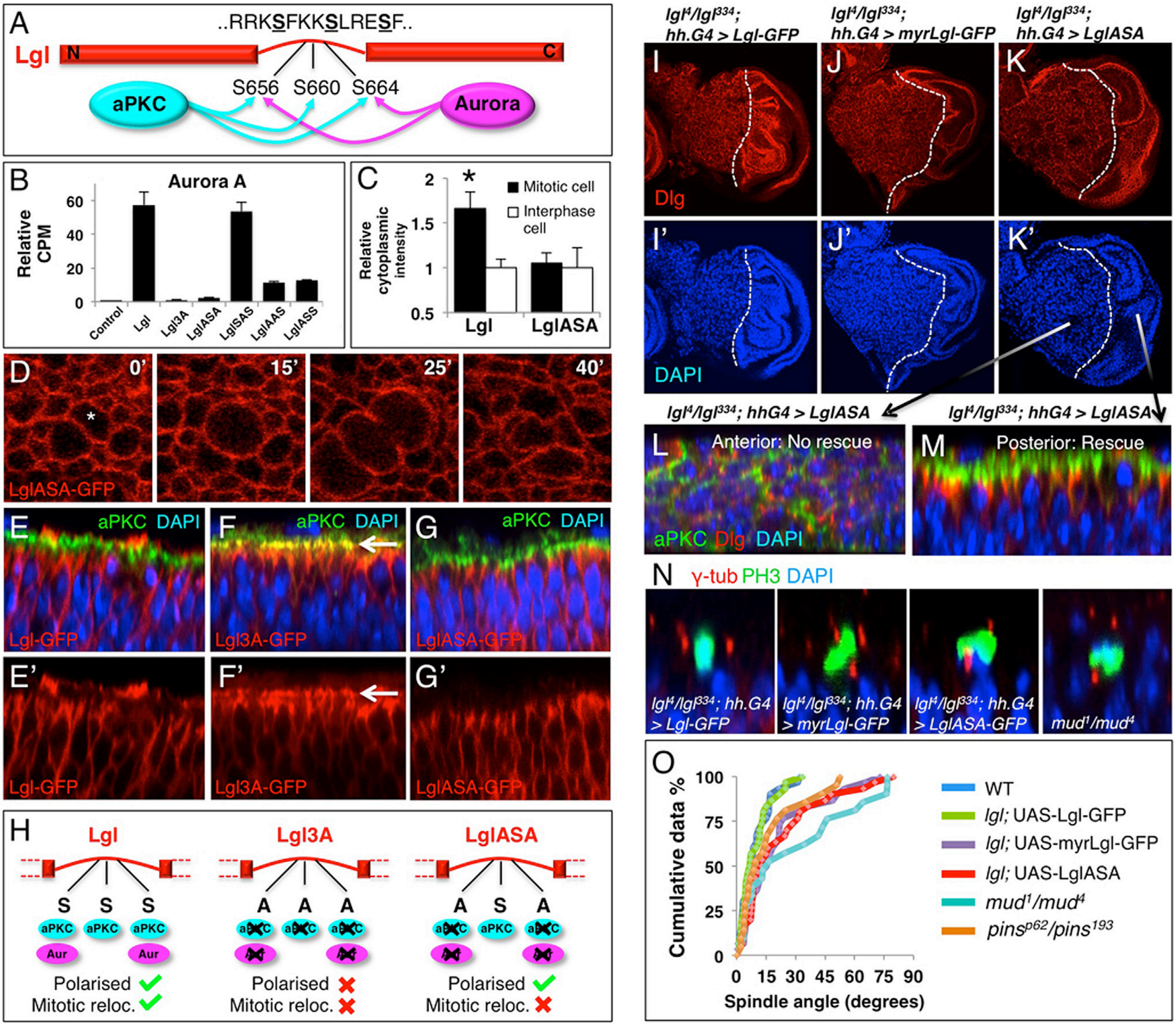

Figure 2. Aurora-Insensitive Lgl Rescues Cell Polarity in the Wing Epithelium but Fails to Relocalize during Mitosis and Disrupts Mitotic Spindle Orientation

(A) Schematic of aPKC and Aurora A/B kinase phosphorylation of Lgl.

(B) Aurora A directly phosphorylates Lgl on S656 and S664 of the tripartite motif, but not S660.

(C) Quantification of cytoplasmic intensity of LglASA-GFP construct compared to Lgl-GFP.

(D) LglASA-GFP does not relocalize to the cytoplasm in mitosis.

(E–G) Lgl-GFP is localized basolaterally and does not overlap with aPKC in wing disc (E). Nonphosphorylatable Lgl3A-GFP spreads apically and colocalizes with aPKC (arrow; F). LglASA-GFP is localized basolaterally and does not overlap with aPKC (G).

(H) Schematic of Lgl, Lgl3A, and LglASA constructs and their respective phosphorylation potential by aPKC or Aurora kinases.

(I) *lgl*^{4}/*lgl*^{334} mutant discs expressing Lgl-GFP in the posterior compartment show a rescue of polarity this compartment.

(J and K) *lgl*^{4}/*lgl*^{334} mutant discs expressing myrLgl-GFP (J) or LglASA-GFP (K) in the posterior compartment show a rescue of cell polarity in this compartment.

(L) Cross-section of the anterior portion of the disc in (K) showing tissue disorganization and lack of polarity.

(M) Cross-section of the posterior portion of the disc in (K) showing normal tissue organization and cell polarization.

(N) Mitotic spindles in wild-type discs are oriented in the plane of the epithelium, whereas clones of *lgl*^{4} mutant cells expressing myrLgl-GFP or LglASA-GFP show misoriented spindles, similar to *pins*$^{p62/193}$ mutants (not shown) or *mud*$^{1/4}$ mutants.

(O) Quantification of mitotic spindle orientation relative to the plane of the epithelium in (N). Low angles reflect planar spindle orientation, whereas high angles reflect more apical-basal spindle orientation. n > 30 for each experiment.

Error bars indicate 1 SD from the mean. See also Figure S2.

then directly responsible for phosphorylating Lgl during mitosis to mediate its removal from the entire plasma membrane and relocalization into the cytoplasm (Figure 4K). This distinction between the action of aPKC and Aurora kinases on Lgl was not originally obvious because they are almost simultaneously activated during asymmetric cell division [11, 14]. Furthermore, the possibility that Aurora directly phosphorylates Lgl was tested using an anti-phospho-Lgl antibody that only recognizes the second serine in the tripartite motif, which is not an Aurora site [14]. Nevertheless, all

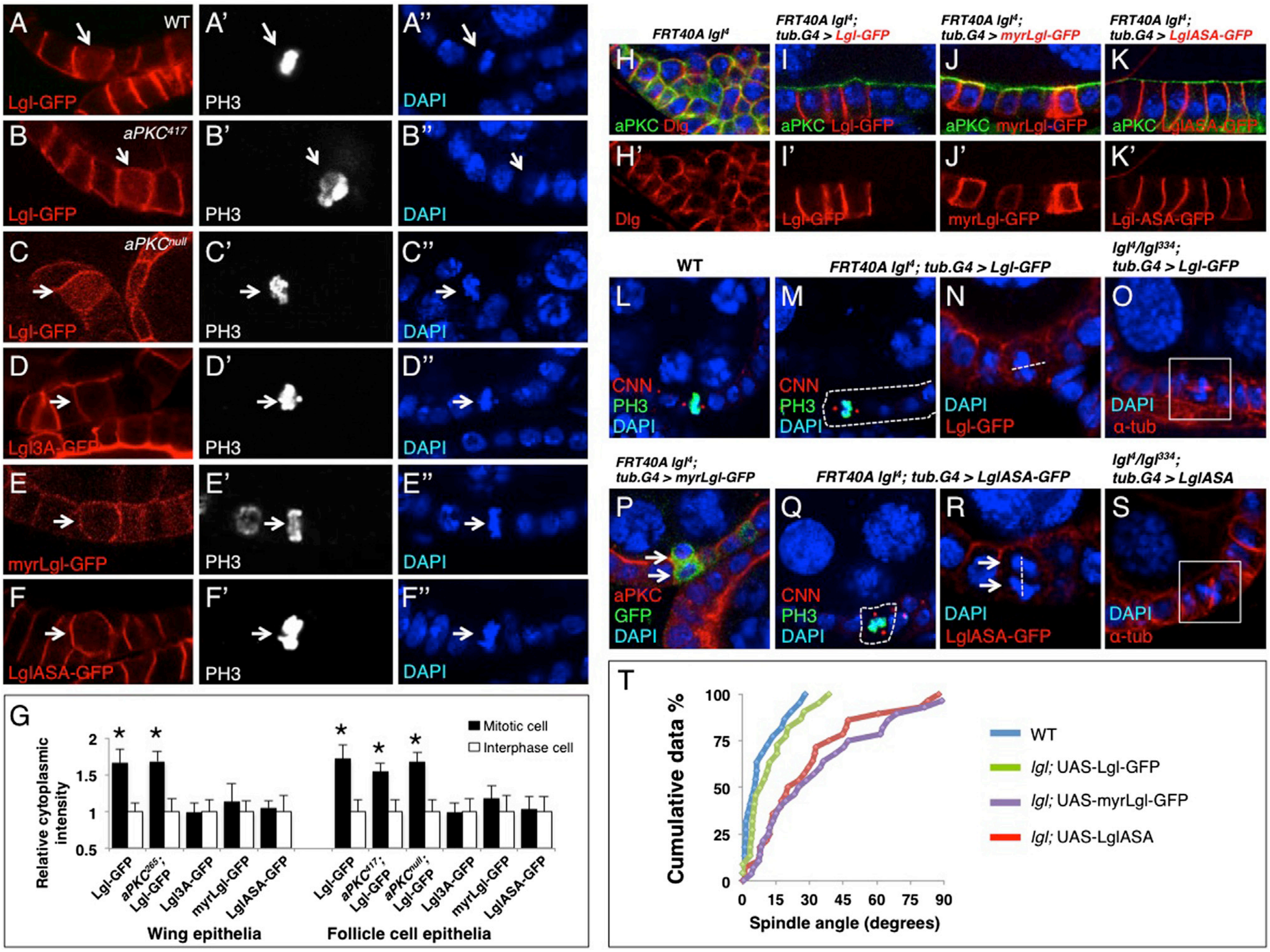

Figure 3. Aurora-Mediated Phosphorylation of Lgl Is Required for Mitotic Spindle Orientation in the Follicle Cell Epithelium

(A) Lgl-GFP relocalizes to the cytoplasm at mitosis in the ovarian follicle cell epithelium.

(B) Relocalization of Lgl to the cytoplasm during mitosis still occurs in *aPKC*^{417} kinase-dead MARCM clones.

(C) Relocalization of Lgl to the cytoplasm during mitosis still occurs in *aPKC*^{K06403}-null mutant MARCM clones.

(D) Phosphomutant Lgl3A-GFP fails to relocalize to the cytoplasm during mitosis.

(E) Membrane-tethered myrLgl-GFP fails to relocalize to the cytoplasm during mitosis.

(F) Aurora-insensitive LglASA-GFP fails to relocalize to the cytoplasm during mitosis.

(G) Quantification of cytoplasmic intensity for (A)–(F). Error bars indicate 1 SD from the mean.

(H–K) Clones of *lgl*^{4} mutant follicle cells show loss of polarity and multilayering (H); this can be rescued by the expression of Lgl-GFP (I), myrLgl-GFP (J), or LglASA-GFP (K).

(L–O) Mitotic spindles in wild-type follicle cell epithelia are oriented in the plane of the epithelium (L). Rescue of spindle orientation is achieved by expression of Lgl-GFP in clones of *lgl*^{4} mutant cells or *lgl*$^{4/334}$ mutant animals (M–O).

(P–S) Mitotic spindles fail to orient in the plane of the epithelium in clones of *lgl*^{4} mutant cells, or *lgl*$^{4/334}$ mutant animals, expressing myrLgl-GFP (P), LglASA-GFP (Q and R), or untagged Lgl-ASA (S).

(T) Quantification of spindle orientation in (L)–(S). n > 20 spindles for each experiment.

other elements of the current model of asymmetric division from remain valid (Figure 4K).

Notably, relocalization of Lgl to the cytoplasm is dispensable for spindle orientation in neuroblasts, presumably due to the neuroblast-specific expression of Inscuteable (Insc), an apical protein that can bind to Pins/LGN and orient the spindle [20, 21] (Figure 4K). After apical recruitment by Insc, Pins/LGN forms a complex with Gαi and Mud/NUMA to orient the spindle in the apical-basal axis [21–24] (Figure 4K). Thus, Pins/LGN does not strictly depend on forming a complex with Dlg for apical-basal spindle orientation in neuroblasts, even though this complex has an essential role in epithelia and other systems [20, 25–27]. In support of this view, ectopic expression of Insc in epithelial cells is sufficient to override the endogenous planar spindle orientation and to re-orient the spindle in the apical-basal axis [28].

In the case of symmetrically dividing epithelial cells, a similar mechanism operates to regulate Lgl localization, which is then essential for spindle orientation in the plane of the epithelium (Figure 4L). aPKC phosphorylation is once again responsible only for polarization of Lgl, while Aurora A/B phosphorylation is responsible only for mitotic relocalization of Lgl to the cytoplasm. This distinction is much more obvious in epithelial cells, because Lgl is constitutively polarized by aPKC activity, whereas Aurora A/B kinases are only activated during mitosis. Relocalization of Lgl has a key role in promoting mitotic

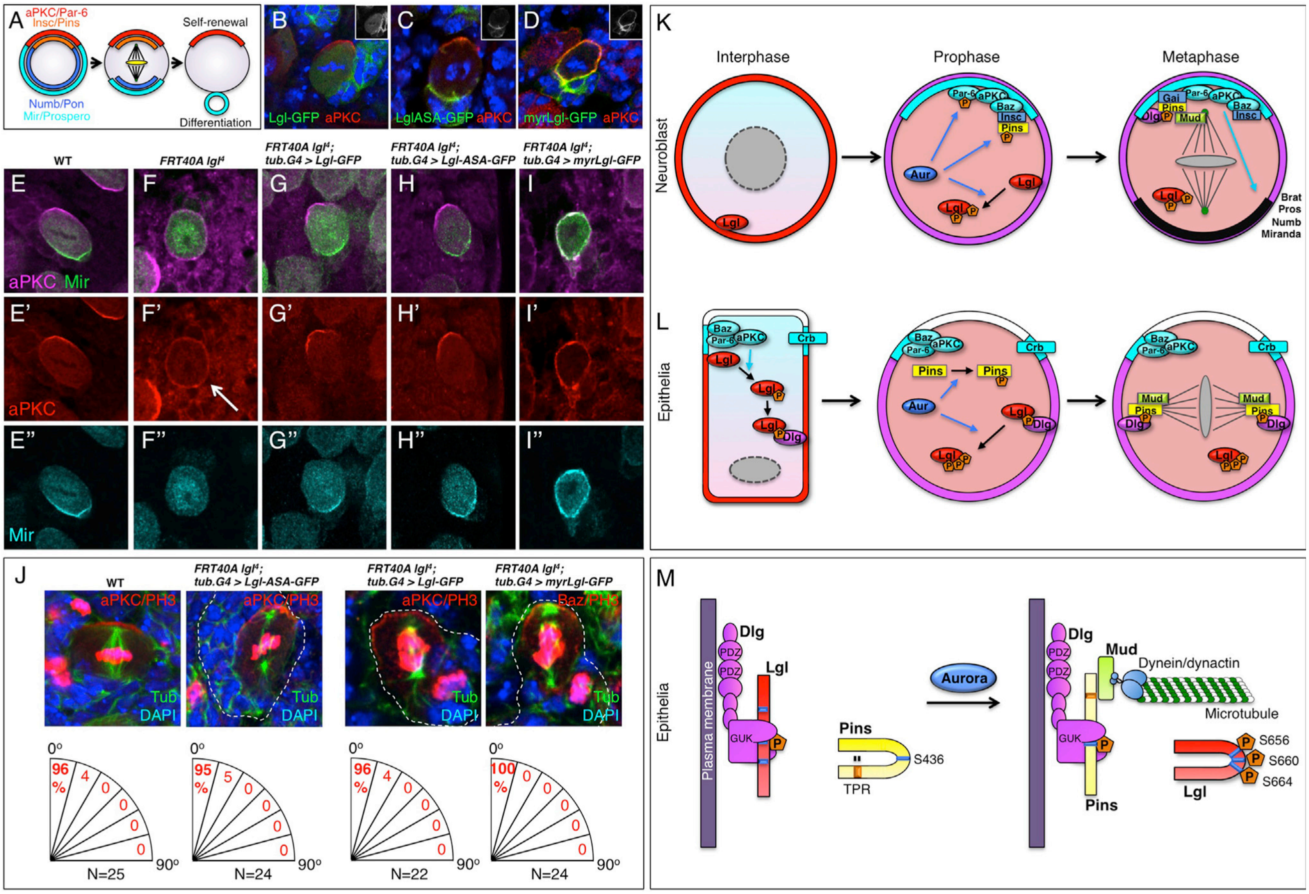

Figure 4. Aurora-Mediated Phosphorylation of Lgl Is Dispensable for Cell Polarity and Mitotic Spindle Orientation in Larval Brain Neuroblasts

(A) Schematic of asymmetric cell division in larval brain neuroblasts.

(B–D) Lgl-GFP is cytoplasmic in metaphase neuroblasts, whereas aPKC is found in an apical crescent (B). LglASA-GFP is polarized to the basal side of the neuroblast and does not affect aPKC localization (C). myrLgl-GFP is localized around the cell cortex and its expression drives aPKC around the cortex (D).

(E–I) aPKC and Miranda form apical and basal crescents, respectively, in metaphase neuroblasts (E). *lgl*[4] mutant neuroblasts show spreading of aPKC and cytoplasmic Miranda (F). *lgl*[4] neuroblasts expressing Lgl-GFP (G) or LglASA-GFP (H) show normal aPKC and Miranda localization. *lgl*[4] neuroblasts expressing myrLgl-GFP show aPKC spreading around the cell cortex and colocalizing with Miranda, indicating that aPKC kinase activity is inhibited by myrLgl-GFP (I).

(J) Quantification of mitotic spindle orientation relative to the apical-basal axis of third-instar larval brain neuroblasts, as marked by either aPKC or Baz localization. Wild-type and *lgl*[4] neuroblasts expressing Lgl-GFP, LglASA-GFP, or myrLgl-GFP all showed show normal spindle orientation. Baz was used to mark the apical-basal axis in the case of myrLgl-GFP because aPKC is no longer restricted apically when this construct is expressed.

(K) Model of asymmetric cell division in neuroblasts. Neuroblasts are not obviously polarized during interphase. Aurora phosphorylates Par-6, Lgl, and Pins at mitosis, and the Baz-aPKC-Par-6 complex becomes apically localized with Insc, which binds to Baz. Phosphorylation of Par-6 promotes aPKC activity and correct segregation of cell fate determinants. Phosphorylation of Lgl on S656 and S664 relocalizes it to the cytoplasm. Pins phosphorylation on S436 and relocalization of Lgl to the cytoplasm promotes Dlg:Pins complex formation, but this complex is not strictly essential for spindle orientation due to Baz-Insc recruitment of Pins and subsequent formation of an apical Gαi:Pins:Mud complex to orient the spindle in the apical-basal axis.

(L and M) Model of symmetric cell division in epithelial cells. Epithelial cells are polarized in interphase, with apical Crb and Baz complexes and basolateral Dlg and Lgl. aPKC-phosphorylated Lgl is removed from the apical membrane and can bind to Dlg at the basolateral membrane in interphase. At mitosis, Aurora phosphorylates Lgl on S656 and S664 to relocalize Lgl to the cytoplasm. Aurora also phosphorylates Pins on S436. These events promote formation of a Dlg:Pins complex, which is essential for spindle orientation within the plane of the epithelium in follicle cells.

spindle orientation in epithelial cells, which are known to depend upon Dlg, Pins/LGN, and Mud/NUMA to orient the spindle in the plane of the epithelium [21, 22, 24–26, 29]. Removal of Lgl from the plasma membrane is presumably necessary for Pins/LGN to bind Dlg and to initiate spindle orientation. Lgl is able to bind directly to the Dlg GUK domain after Lgl has been phosphorylated by aPKC [30]. Efficient binding of Pins/LGN to the Dlg GUK domain would therefore require removal of Lgl from the membrane. In addition, Aurora A phosphorylation of Ser436 of Pins/LGN was proposed to be essential for it to interact with the Dlg GUK domain and to orient the mitotic spindle [27]. Thus, these data indicate that Aurora kinases control the onset of spindle orientation by phosphorylating both Lgl and Pins/LGN to disrupt any Lgl-Dlg binding and to induce the Pins/LGN-Dlg interaction in epithelial cells (Figure 4M). Notably, the *pins* mutant phenotype is weaker in imaginal discs than follicle cells, which suggests the existence of another Dlg-binding protein that may act in parallel with Pins in this tissue.

In conclusion, the Lgl tumor suppressor is regulated by similar mechanisms in both symmetric and asymmetric divisions. The aPKC kinase controls polarization of Lgl, whereas

the Aurora A and B kinases control mitotic relocalization of Lgl to the cytoplasm in both epithelia and neuroblasts. Mitotic relocalization of Lgl is essential to promote mitotic spindle orientation in epithelia, but not in neuroblasts. Future work on Lgl and its role as a tumor suppressor should consider not only its function at the cell cortex but also its role in regulating the mitotic spindle. Finally, Aurora kinases are known to be overexpressed in many human cancers, and the Lgl protein may be an important target for Auroras in promoting tumor formation and progression.

Experimental Procedures

Drosophila Genetics

The following strains were used: *FRT42B* $aPKC^{psu141}$, *FRT42B* $aPKC^{psu417}$, *FRT42B* $aPKC^{psu265}$ [31]; *FRT82B* $aurA^{14641}$ [6]; *Crb-GFP* [32]; *UAS-Baz-mCherry* [33]; *UAS-Lgl-GFP, UAS-Lgl3A-GFP* [34]; *UAS-Dlg-GFP* [35]; *UAS-Par6-GFP* [36]; *Scrib-GFP* [37]; *UAS-AurA-GFP* [38]; *FRT40A* lgl^{4}, $aurA^{87Ac-3}$, lgl^{334}, *FRT42B* $aPKC^{K06403}$, *Df(3R)Exel6163*, dlg^{1}, dlg^{M52} (Bloomington *Drosophila* Stock Centre); and *UAS-AurB-RNAi* (KK library). *UAS-myrLgl-GFP*, *UAS-LglASA*, and *UAS-LglASA-GFP* constructs were generated for this study. Additional methods are described in the Supplemental Experimental Procedures.

Supplemental Information

Supplemental Information includes Supplemental Experimental Procedures, three figures, and three movies and can be found with this article online at http://dx.doi.org/10.1016/j.cub.2014.10.052.

Acknowledgments

This work was funded by Cancer Research UK and by a Wellcome Trust Investigator Award to B.J.T.

Received: June 23, 2014
Revised: September 22, 2014
Accepted: October 21, 2014
Published: December 4, 2014

References

1. Bryant, P.J., and Schubiger, G. (1971). Giant and duplicated imaginal discs in a new lethal mutant of Drosophila melanogaster. Dev. Biol. *24*, 233–263.
2. Mechler, B.M., McGinnis, W., and Gehring, W.J. (1985). Molecular cloning of lethal(2)giant larvae, a recessive oncogene of Drosophila melanogaster. EMBO J. *4*, 1551–1557.
3. Jacob, L., Opper, M., Metzroth, B., Phannavong, B., and Mechler, B.M. (1987). Structure of the l(2)gl gene of Drosophila and delimitation of its tumor suppressor domain. Cell *50*, 215–225.
4. Lützelschwab, R., Klämbt, C., Rossa, R., and Schmidt, O. (1987). A protein product of the Drosophila recessive tumor gene, l (2) giant gl, potentially has cell adhesion properties. EMBO J. *6*, 1791–1797.
5. Bilder, D., Li, M., and Perrimon, N. (2000). Cooperative regulation of cell polarity and growth by Drosophila tumor suppressors. Science *289*, 113–116.
6. Lee, C.Y., Andersen, R.O., Cabernard, C., Manning, L., Tran, K.D., Lanskey, M.J., Bashirullah, A., and Doe, C.Q. (2006). Drosophila Aurora-A kinase inhibits neuroblast self-renewal by regulating aPKC/Numb cortical polarity and spindle orientation. Genes Dev. *20*, 3464–3474.
7. Lee, C.Y., Robinson, K.J., and Doe, C.Q. (2006). Lgl, Pins and aPKC regulate neuroblast self-renewal versus differentiation. Nature *439*, 594–598.
8. Tepass, U. (2012). The apical polarity protein network in Drosophila epithelial cells: regulation of polarity, junctions, morphogenesis, cell growth, and survival. Annu. Rev. Cell Dev. Biol. *28*, 655–685.
9. St Johnston, D., and Ahringer, J. (2010). Cell polarity in eggs and epithelia: parallels and diversity. Cell *141*, 757–774.
10. Thompson, B.J. (2013). Cell polarity: models and mechanisms from yeast, worms and flies. Development *140*, 13–21.
11. Betschinger, J., Mechtler, K., and Knoblich, J.A. (2003). The Par complex directs asymmetric cell division by phosphorylating the cytoskeletal protein Lgl. Nature *422*, 326–330.
12. Hutterer, A., Betschinger, J., Petronczki, M., and Knoblich, J.A. (2004). Sequential roles of Cdc42, Par-6, aPKC, and Lgl in the establishment of epithelial polarity during Drosophila embryogenesis. Dev. Cell *6*, 845–854.
13. Betschinger, J., Eisenhaber, F., and Knoblich, J.A. (2005). Phosphorylation-induced autoinhibition regulates the cytoskeletal protein Lethal (2) giant larvae. Curr. Biol. *15*, 276–282.
14. Wirtz-Peitz, F., Nishimura, T., and Knoblich, J.A. (2008). Linking cell cycle to asymmetric division: Aurora-A phosphorylates the Par complex to regulate Numb localization. Cell *135*, 161–173.
15. Carvalho, C.A., Moreira, S., Ventura, G., Sunkel, C.E., and Morais-de-Sá, E. (2014). Aurora A triggers Lgl cortical release during symmetric division to control planar spindle orientation. Curr. Biol. Published online December 4, 2014. http://dx.doi.org/10.1016/j.cub.2014.10.053.
16. Huang, J., Zhou, W., Dong, W., Watson, A.M., and Hong, Y. (2009). From the cover: directed, efficient, and versatile modifications of the Drosophila genome by genomic engineering. Proc. Natl. Acad. Sci. USA *106*, 8284–8289.
17. Carmena, M., Ruchaud, S., and Earnshaw, W.C. (2009). Making the Auroras glow: regulation of Aurora A and B kinase function by interacting proteins. Curr. Opin. Cell Biol. *21*, 796–805.
18. Zeitlin, S.G., Barber, C.M., Allis, C.D., and Sullivan, K.F. (2001). Differential regulation of CENP-A and histone H3 phosphorylation in G2/M. J. Cell Sci. *114*, 653–661.
19. Kunitoku, N., Sasayama, T., Marumoto, T., Zhang, D., Honda, S., Kobayashi, O., Hatakeyama, K., Ushio, Y., Saya, H., and Hirota, T. (2003). CENP-A phosphorylation by Aurora-A in prophase is required for enrichment of Aurora-B at inner centromeres and for kinetochore function. Dev. Cell *5*, 853–864.
20. Siegrist, S.E., and Doe, C.Q. (2005). Microtubule-induced Pins/Galphai cortical polarity in Drosophila neuroblasts. Cell *123*, 1323–1335.
21. Siller, K.H., and Doe, C.Q. (2009). Spindle orientation during asymmetric cell division. Nat. Cell Biol. *11*, 365–374.
22. Gonzalez, C. (2007). Spindle orientation, asymmetric division and tumour suppression in Drosophila stem cells. Nat. Rev. Genet. *8*, 462–472.
23. Mapelli, M., and Gonzalez, C. (2012). On the inscrutable role of Inscuteable: structural basis and functional implications for the competitive binding of NuMA and Inscuteable to LGN. Open Biol. *2*, 120102.
24. Bergstralh, D.T., Haack, T., and St Johnston, D. (2013). Epithelial polarity and spindle orientation: intersecting pathways. Philos. Trans. R. Soc. Lond. B Biol. Sci. *368*, 20130291.
25. Bergstralh, D.T., Lovegrove, H.E., and St Johnston, D. (2013). Discs large links spindle orientation to apical-basal polarity in Drosophila epithelia. Curr. Biol. *23*, 1707–1712.
26. Bellaïche, Y., Radovic, A., Woods, D.F., Hough, C.D., Parmentier, M.L., O'Kane, C.J., Bryant, P.J., and Schweisguth, F. (2001). The Partner of Inscuteable/Discs-large complex is required to establish planar polarity during asymmetric cell division in Drosophila. Cell *106*, 355–366.
27. Johnston, C.A., Hirono, K., Prehoda, K.E., and Doe, C.Q. (2009). Identification of an Aurora-A/PinsLINKER/Dlg spindle orientation pathway using induced cell polarity in S2 cells. Cell *138*, 1150–1163.
28. Egger, B., Boone, J.Q., Stevens, N.R., Brand, A.H., and Doe, C.Q. (2007). Regulation of spindle orientation and neural stem cell fate in the Drosophila optic lobe. Neural Dev. *2*, 1.
29. Nakajima, Y., Meyer, E.J., Kroesen, A., McKinney, S.A., and Gibson, M.C. (2013). Epithelial junctions maintain tissue architecture by directing planar spindle orientation. Nature *500*, 359–362.
30. Zhu, J., Shang, Y., Wan, Q., Xia, Y., Chen, J., Du, Q., and Zhang, M. (2014). Phosphorylation-dependent interaction between tumor suppressors Dlg and Lgl. Cell Res. *24*, 451–463.
31. Kim, S., Gailite, I., Moussian, B., Luschnig, S., Goette, M., Fricke, K., Honemann-Capito, M., Grubmüller, H., and Wodarz, A. (2009). Kinase-activity-independent functions of atypical protein kinase C in Drosophila. J. Cell Sci. *122*, 3759–3771.
32. Huang, J., Zhou, W., Dong, W., and Hong, Y. (2009). Targeted engineering of the Drosophila genome. Fly (Austin) *3*, 274–277.
33. Pope, K.L., and Harris, T.J. (2008). Control of cell flattening and junctional remodeling during squamous epithelial morphogenesis in Drosophila. Development *135*, 2227–2238.

34. Tian, A.G., and Deng, W.M. (2008). Lgl and its phosphorylation by aPKC regulate oocyte polarity formation in Drosophila. Development *135*, 463–471.
35. Koh, Y.H., Popova, E., Thomas, U., Griffith, L.C., and Budnik, V. (1999). Regulation of DLG localization at synapses by CaMKII-dependent phosphorylation. Cell *98*, 353–363.
36. David, D.J., Tishkina, A., and Harris, T.J. (2010). The PAR complex regulates pulsed actomyosin contractions during amnioserosa apical constriction in Drosophila. Development *137*, 1645–1655.
37. Venken, K.J., Schulze, K.L., Haelterman, N.A., Pan, H., He, Y., Evans-Holm, M., Carlson, J.W., Levis, R.W., Spradling, A.C., Hoskins, R.A., and Bellen, H.J. (2011). MiMIC: a highly versatile transposon insertion resource for engineering Drosophila melanogaster genes. Nat. Methods *8*, 737–743.
38. Berdnik, D., and Knoblich, J.A. (2002). Drosophila Aurora-A is required for centrosome maturation and actin-dependent asymmetric protein localization during mitosis. Curr. Biol. *12*, 640–647.

Current Biology, Volume *25*
Supplemental Information

Aurora Kinases Phosphorylate Lgl to Induce Mitotic Spindle Orientation in *Drosophila* Epithelia

Graham P. Bell, Georgina C. Fletcher, Ruth Brain, and Barry J. Thompson1

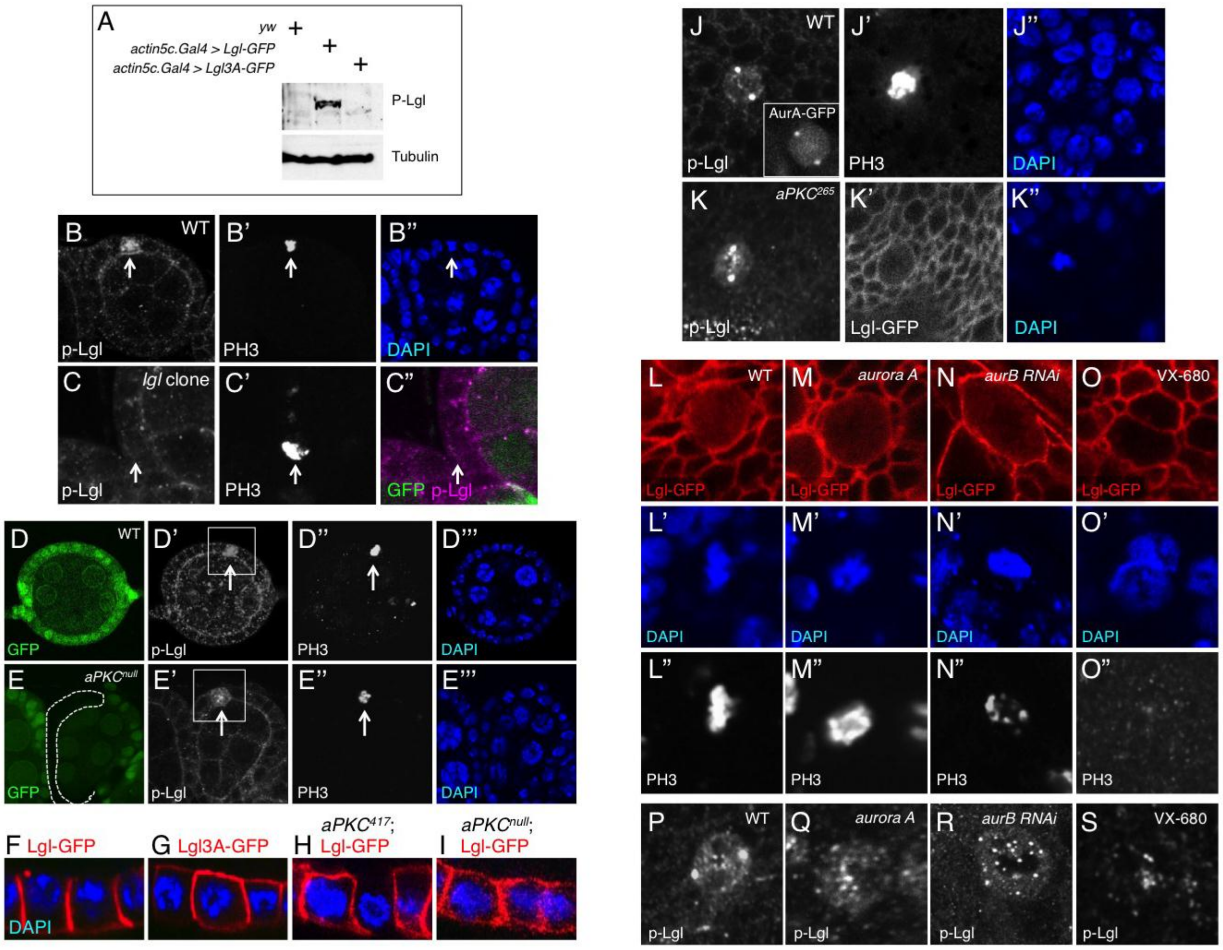

Figure S1. Mitotic phosphorylation of Lgl depends on Aurora A/B kinases, while aPKC kinase activity is only required to polarise Lgl during interphase.

(A) A phospho-Lgl antibody is detectable by Western blotting upon expression of Lgl-GFP, but not Lgl3A-GFP or in *yw* specimens.
(B-C) Phospho-Lgl strongly accumulates in the cytoplasm of mitotic cells in the follicle cell epithelium of control (B) but not lgl^{4} mutant clones (C).
(D-E) Phospho-Lgl strongly accumulates in the cytoplasm of mitotic cells in both control (D) and $aPKC^{K06403}$ null mutant (E) follicle cells.
(F) Lgl-GFP control showing normal polarisation in the follicle cell epithelium.
(G) Lgl3A-GFP is not polarised and localises around the entire plasma membrnane.
(H) Lgl-GFP is no longer polarised in $aPKC^{417}$ kinase-dead mutant MARCM clones.
(I) Lgl-GFP is no longer polarised in $aPKC^{K06403}$ null mutant MARCM clones.
(J-K) Phospho-Lgl accumulates in the cytoplasm of mitotic cells in the wing imaginal discs of both control (J) and $aPKC^{265}$ kinase-dead mutant MARCM clones (K). Note that AuroraA-GFP localisation is almost identical to p-Lgl antibody staining (inset).
(L-O) Lgl can still relocalise to the cytoplasm in control (L), $aurA^{87Ac\text{-}3}$/*Df* mutants (M) or *en.Gal4 UAS-aurB-RNAi* (N), but not upon treatment with the dual Aurora kinase inhibitor VX-680 (O). PH3 staining was not affected by mutation of *aurA* (M), but was strongly reduced in both *aurB* RNAi (N) and VX-680 treated discs (O).
(P-S) Phospho-Lgl accumulation in mitosis (P) can still be detected in either $aurA^{87Ac\text{-}3}$/*Df* mutants (Q) or *en.Gal4 UAS-aurB-RNAi* (N), but not upon treatment with the dual Aurora kinase inhibitor VX-680 (S). P, Q and S are the same cell as in L, M and O.

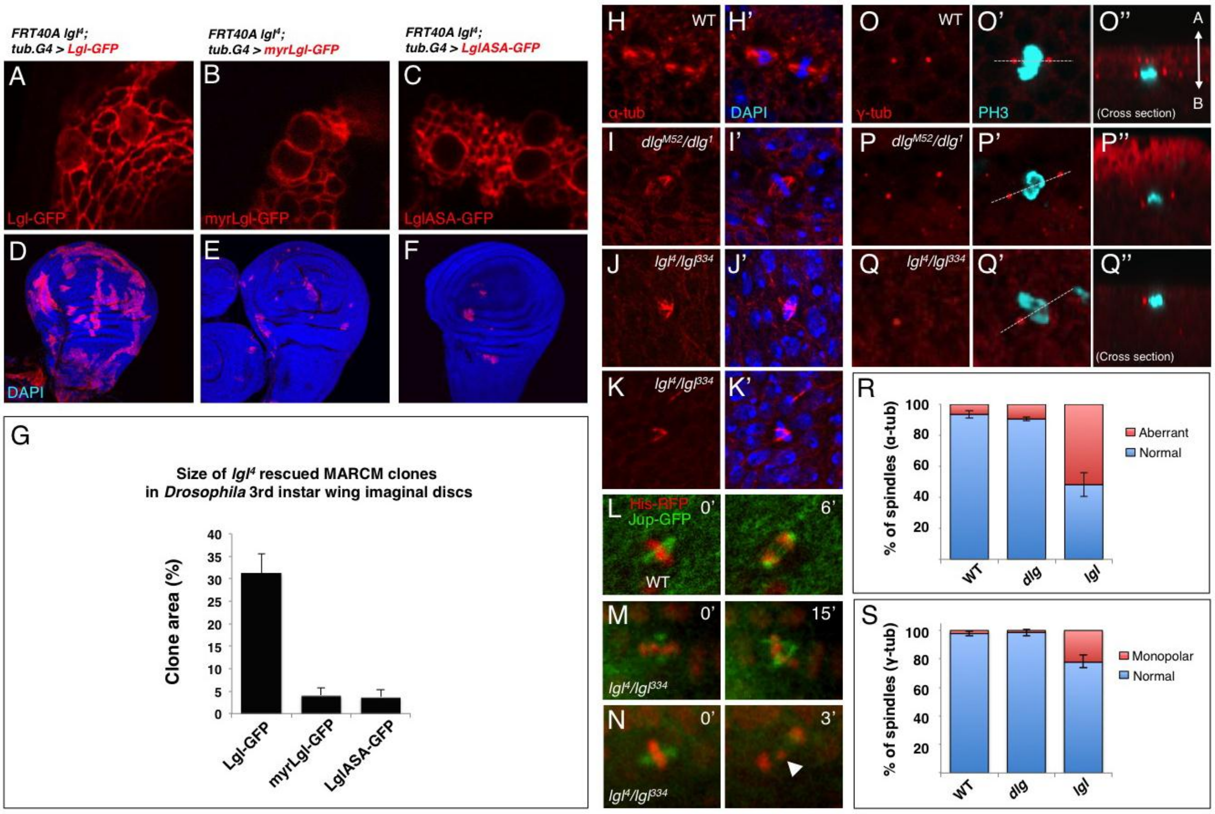

Figure S2. A mitotic role for Lgl in clonal growth and spindle formation in the wing imaginal disc epithelium.

(A-G) MARCM clones of *lgl*^{4} mutant cells expressing Lgl-GFP (A), myrLgl-GFP (B) or LglASA-GFP (C). Note that myr-Lgl-GFP and LglASA-GFP do not relocalise efficiently to the cytoplasm at mitosis. Whilst clones of *lgl*^{4} mutant cells expressing Lgl-GFP grow normally (D), clones of *lgl*^{4} mutant cells expressing myrLgl-GFP (E) or LglASA-GFP (F) grow to a small size, suggesting a mitotic role for Lgl that is required to prevent apoptosis or promote cell proliferation. (G) Quantification of clone sizes in D-F.
(H-K) Mitotic spindles form normally in wild type (H) and *dlg*^{M52}/*dlg*^{1} (I) wing imaginal discs. Mitotic spindles form abnormally in *lgl*^{4}/*lgl*^{334} mutant wing imaginal discs (J,K). α-tubulin (red) and DAPI (blue) are stained.
(L-N) Live imaging of wild type (L) or *lgl*^{4}/*lgl*^{334} (M, N) mutant discs reveals similar defects in spindle organisation to fixed samples. Note the similarity in spindle phenotype between (K) and (M). Note the unsuccessful chromosome segregation in (N).
(O-Q) Mitotic spindles in wild type tissues are always bipolar, and are oriented parallel to the plane of the tissue (O). *dlg*^{M52}/*dlg*^{1} mutant discs show spindles misaligned with the plane of the tissue, but no defects in centrosome number (P). *lgl*^{4}/*lgl*^{334} mutant discs show some monopolar spindles, in addition to defects in spindle orientation (Q).
(R-S) Quantification of mitotic spindle defects in wild type, *dlg* mutant and *lgl* mutant discs by α-tubulin (R) or γ tubulin (S) staining.

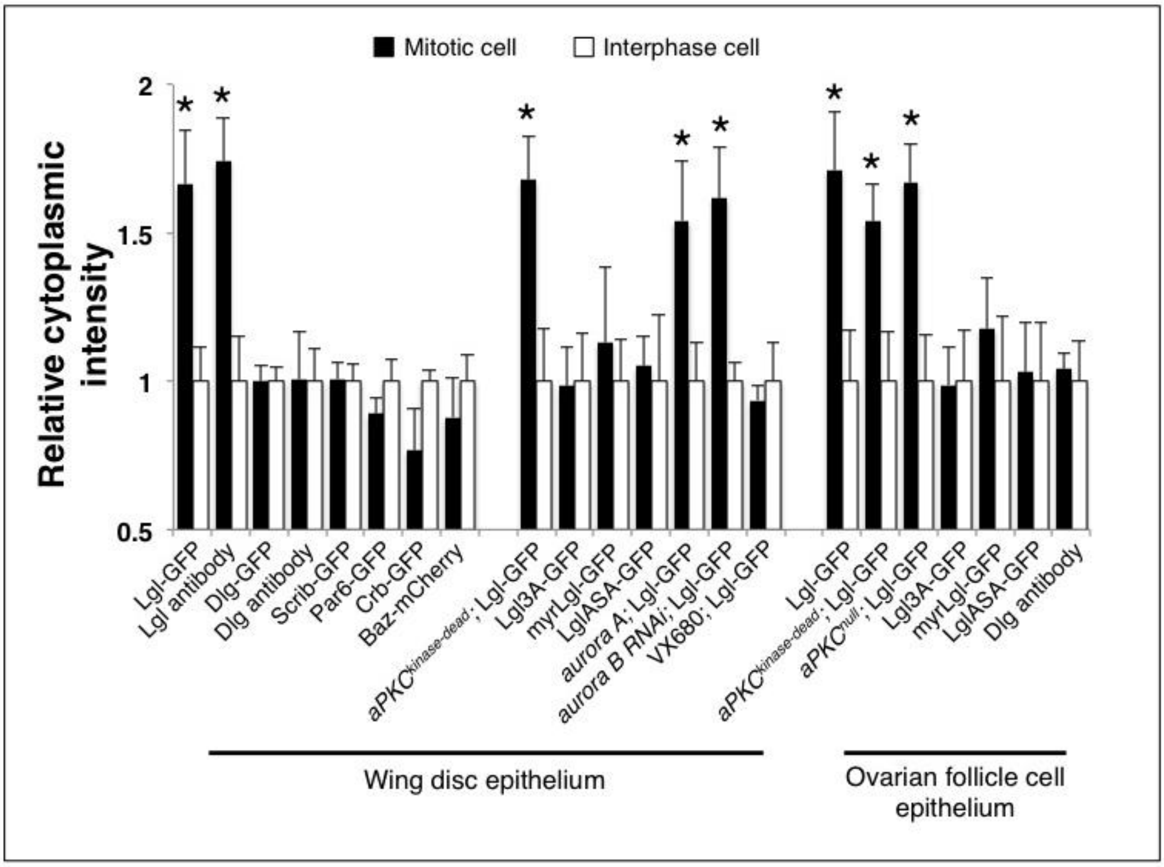

Figure S3. Quantification of intensity measurements.

Supplemental Experimental Procedures

Drosophila genetics

Transgenes driven by a UAS-promoter were induced by crossing lines to the *MS1096.Gal4, en.Gal4,* or *hh.Gal4* drivers for wing disc expression; either *tub.Gal4* or *tj.Gal4* for follicle cell expression; *wor.Gal4* for neuroblasts; and *actin5c.Gal4* for whole animal expression. Mitotic clones were generated using the FLP/FRT system by heat-shocking flies at 37 degrees for 1hr during early larval development, and marked negatively (absence of GFP) or positively (presence of GFP; MARCM). Expression of *UAS-Lgl-GFP, UAS-myrLgl-GFP,* or *UAS-LglASA-GFP* in *FRT* wild-type or *FRT* mutant backgrounds was achieved using the MARCM system. Clones were induced at the same time in cultures of controlled density in each experiment. For wing disc or brain clonal analysis, third instar larvae were dissected; for follicle cell analysis, heat-shocked larvae were grown to adult, and females dissected approximately 4 days after eclosion.

Antibody staining

Wing imaginal discs or brains dissected from third instar larvae, or ovaries dissected from adult females, were fixed for 30 mins in cold PBS containing 4% paraformaldehyde. Samples were washed in PBS supplemented with 0.1% Triton-X100 (PBT), blocked with 0.1% Bovine Serum Albumin (PBT + BSA) (and 5% Normal Goat Serum for ovaries), and stained with primary and fluorescently conjugated secondary antibodies.

Primary antibodies used were:

Rabbit anti-phospho LLGL1/2 (p-Lgl) (1:250, Abgent), mouse anti-phosphohistone H3 (PH3) (1:1000, Abcam), rabbit anti-phosphohistone H3 (PH3) (1:1000, Millipore), mouse anti-α tubulin (1:500, Sigma), mouse anti-Dlg (1:250 DSHB), rabbit anti-aPKC (1:250, Santa Cruz), rabbit anti-γ tubulin (1:500, Sigma), mouse anti-Miranda (1:50, gift from A. Gould), mouse anti-GFP (1:250, Roche), rabbit anti-

centrosomin (CNN) (1:1000, gift from J. Raff).

Secondary antibodies (all from Molecular Probes, Invitrogen), were used at 1:500 for 2 hours prior to multiple washes in PBT and staining with DAPI at 1µg/ml for 10-minutes, before mounting on slides in Vectashield (Vector labs). Images were taken on a Leica SP5 confocal and processed with Adobe Photoshop and ImageJ.

Live imaging
Live imaging of *ex vivo* cultured wing discs was performed with a Zeiss 780 confocal microscope. Briefly, discs were cultured in Shields and Sang M3 media (Sigma) containing 2% fetal bovine serum, 10 µg/mL streptomycin/penicillin (Invitrogen), 10 mU/L insulin, 0.1 µg/mL ecdysone (Sigma) and 2.5% methyl cellulose (Sigma), and imaged in a 35mm-Fluorodish. *Z*-stacks were taken at 1-µm intervals, with total thickness of 10–30 µm. *Z*-stacks were typically scanned at 2-min intervals for up to 3 h. Images were projected and time points were collated using Zen software.

For drug treatment, inverted larvae were incubated in culture media as described above (without methyl cellulose) with 2µM VX-680/Tozasertib (Selleck BioChem) for 30-60 minutes, then transferred to media as described above for imaging.

In vitro kinase assay
For *in vitro* kinase assays, HPLC purified peptide substrates were incubated with either 200ng recombinant human Aurora A (Promega) or 80ng recombinant human Aurora B (Promega) for 30 min at 30°C in Reaction Buffer A (Promega) (200mM Tris-HCL (pH 7.5), 100mM $MgCl_2$, 0.5 µg/µl BSA, 50µM DTT) containing 10µM cold adenosine triphosphate (ATP) and 3µCi of ϒ-P32 ATP (Perkin Elmer). Samples were blotted on P81 phosphocellulose paper (Millipore) and washed 3 x 10 min in 1% phosphoric acid, then once in acetone. Incorporation of γ-P^{32} was quantified in counts per minute (cpm) by liquid scintillation (Beckman LS 6500).

Peptides used in this study were:

Lgl	LSRRKSFKKSLRESFRKLR (2422.91 Da)
Lgl 3A (S656A, S660A, S664A)	LSRRKAFKKALREAFRKLR (2374.91 Da)
Lgl ASA (S656A, S664A)	LSRRKAFKKSLREAFRKLR (2390.91 Da)
Lgl SAS (S660A)	LSRRKSFKKALRESFRKLR (2406.91 Da)
Lgl AAS (S656A, S660A)	LSRRKAFKKALRESFRKLR (2390.91 Da)
Lgl ASS (S656A	LSRRKAFKKSLRESFRKLR (2406.91 Da)
CENP-A	MGPRRRSRKPEAPRRRSPSP (2374.77 Da)
CENP-A S7A	MGPRRRARKPEAPRRRSPSP (2358.77 Da)

Peptides were diluted with deionised water to working dilutions (1mg/ml) and stored at -20°C.

Western Blotting
Actin5c.Gal4/UAS-Lgl-GFP or *Actin5c.Gal4/UAS-Lgl3A-GFP* third instar larvae were dissected in cold PBS, and homogenised in sample buffer (50mM Tris pH7.5, 150mM NaCL, 1% Triton X-100, 1µM EGTA) supplemented with protease inhibitor cocktail (Roche), and phosphatase inhibitor cocktail (Roche) where appropriate. Samples were incubated for 30 minutes: with 2000 units/ml λ protein phosphatase (New England Biolabs) at 30°C, with 2µM VX-680, or on ice. SDS sample buffer and reducing agent (NuPage, Invitrogen) were added, and samples heated at 70°C for 10 minutes before SDS Page and Western Blotting (NuPage, Invitrogen). Nitrocellulose membranes were probed with rabbit anti-phospho-LLGL1/2 (1:100, Abgent) and mouse anti-Tubulin (1:1000, DSHB), and after several washes in TBST, probed with HRP-conjugated secondary antibodies (1:10,000, Thermo Scientific), with proteins detected by chemiluminescence.
